# New Appliances and Things Medical

**Published:** 1897-08-28

**Authors:** 


### NEW APPLIANCES AND THINGS MEDICAL.

[We shall be glad to receive, at our Office, 28 & 29, Southampton Street, Strand, London, W.C., from the manufacturers, specimens of all new preparations and appliances which may be brought out from time to time.]

### KOLLOX WINE.

(The Kollox Wine Company, 69, Mark Lane, E.C.)

This most agreeable preparation has recently been submitted to our notice, and such trial as we have made of it thoroughly confirms the opinion that we formed of it from a perusal of the method of preparation and the character of the ingredients. The basis of this preparation is port wine, which we believe to be excellent quality, and with the wine are combined a specially prepared fluid beef and a certain proportion of the proximate principles of kola, which have known and useful therapeutic value. The result of this combination is that in one and the same preparation there is a most agreeable blending of stimulant, tonic, and nutritive bodies; hence Kollox may be justly described as a "tonic food." For the convalescent, the over-worked in body or mind, and the debilitated this new preparation has undoubted virtues for its recommendation. As for quantity it should be taken as is usually the case with port wine, in doses of a wine-glass two or three times a day.

### IZAL PREPARATIONS.

(Newton, Chambers, and Co., Thorncliffe, Sheffield, England.)

We have more than once had occasion to make mention in the columns of this journal of the various preparations manufactured by the proprietors of the disinfectant Izal. We have recently received samples of Izal bar, scented, toilet, medical, and soft soaps, Izal cream, ointment, embrocation, lozenges, and tooth powder, and after somewhat prolonged and careful examination we are pleased to refer them to the notice of our readers as preparations of undoubted value. There is no antiseptic which, in our mind, can be advantageously compared to Izal; that is to say, as far as the three following important points are concerned: (1) Germicidal properties; (2) non-poisonous properties; (3) the ease with which it can be combined with toilet and other preparations for internal and external use; and (4) its comparatively agreeable smell and taste. We have no space to enter here into details, but we would specially call attention to three preparations: (1) Izal tooth powder, a dental preparation of peculiarly valuable antiseptic properties and cleansing powers; (2) Izal cream, a very pleasant and emollient application for the skin, and particularly to be recommended for rubbing into the skin during the stages of desquamation after scarlet or other specific fever; (3) Izal ointment, an ointment of considerable value for the treatment of open wounds, burns, scalds, &c., when antiseptic treatment is of importance. The chief value of the Izal preparations is, perhaps, due to the fact that they are domestic and household medicaments, which can be safely applied in all cases without medical advice or on an emergency.

### CREPE BANDAGES.

(W. Gimber, 6, Harders Road, Peckham, London, S.E.)

These new bandages strike us as being particularly practical and useful, and were they a little cheaper in price we would be prepared to predict that they would be universally used in surgical practice, except in cases where great rigidity and support are required. They are very easy of application, even by the inexperienced; they are remarkably comfortable, wonderfully light and porous. We intend to give them an extensive trial in cases of varicose veins, and for the support of weak or diseased joints, as also for the keeping in place of dressings to the scalp or face, owing to their extreme softness and elasticity. We believe that they will be much appreciated by patients. Price 15s. and 18s. per dozen.

### CALVERT'S CARBOLIC PREPARATIONS.

(F. C. Calvert and Co., Manchester.)

We have recently received a number of samples of the more important of Calvert's antiseptic and disinfectant preparations. The list includes a variety of preparations for the cleaning and preservation of the teeth, including carbolic tooth powder and paste, and a very agreeable antiseptic wash for the teeth and mouth. Each of these in its own particular way is an invaluable medium for the maintenance of the teeth in a healthy and sound condition.

A very useful form in which carbolic acid is prepared by this firm is that which is called the Carbolic Safety Tablets. In each of the tablets carbolic acid is present to the extent of 50 per cent., and with this knowledge a solution of the acid may be made at an instant's notice of any required strength, and may be carried with convenience in the surgeon's bag without any of the attendant dangers inseparable from the carriage of the strong liquid forms. Calvert's household and toilet soaps, as well as the various disinfecting powders, each with its particular percentage of carbolic acid, are too well known to require further mention. Some of the newer productions of this firm have been examined by us with results that show that the same care and skill have been employed in their manufacture, that have made their older and better-known preparations famous.

